# Effect of Intermittent vs. Continuous Energy Restriction on Visceral Fat: Protocol for The Healthy Diet and Lifestyle Study 2 (HDLS2)

**DOI:** 10.3390/nu16101478

**Published:** 2024-05-14

**Authors:** Michelle Y. Lewis, Kim Yonemori, Alison Ross, Lynne R. Wilkens, John Shepherd, Kevin Cassel, Andrew Stenger, Christoph Rettenmeier, Unhee Lim, Carol Boushey, Loïc Le Marchand

**Affiliations:** 1 Population Sciences in the Pacific Program, University of Hawai’i Cancer Center, Honolulu, HI 96813, USA; 2 MRI Research Center, John A. Burns School of Medicine, University of Hawai’i, Honolulu, Hi 96813, USA

**Keywords:** obesity, intermittent energy restriction (IER), daily energy restriction, Mediterranean diet (MED), visceral adipose tissue (VAT), metabolic health, randomized control trial (RCT), body composition, dietary behavioral intervention, metabolic dysfunction-associated fatty liver disease (MAFLD)

## Abstract

Obesity in the United States and Western countries represents a major health challenge associated with an increased risk of metabolic diseases, including cardiovascular disease, hypertension, diabetes, and certain cancers. Our past work revealed a more pronounced obesity–cancer link in certain ethnic groups, motivating us to develop a tailored dietary intervention called the Healthy Diet and Lifestyle 2 (HDLS2). The study protocol is described herein for this randomized six-month trial examining the effects of intermittent energy restriction (5:2 Diet) plus the Mediterranean dietary pattern (IER + MED) on visceral adipose tissue (VAT), liver fat, and metabolic biomarkers, compared to a standard MED with daily energy restriction (DER + MED), in a diverse participant group. Using MRI and DXA scans for body composition analysis, as well as metabolic profiling, this research aims to contribute to nutritional guidelines and strategies for visceral obesity reduction. The potential benefits of IER + MED, particularly regarding VAT reduction and metabolic health improvement, could be pivotal in mitigating the obesity epidemic and its metabolic sequelae. The ongoing study will provide essential insights into the efficacy of these energy restriction approaches across varied racial/ethnic backgrounds, addressing an urgent need in nutrition and metabolic health research. Registered Trial, National Institutes of Health, ClinicalTrials.gov (NCT05132686).

## 1. Introduction

In the United States and many Western countries, obesity has grown into a significant health challenge. Ten years ago, no U.S. state had an adult obesity prevalence rate higher than 34%. Obesity rates have progressively increased, and in 2022, as many as 22 states were reporting rates ≥ 35% [1]. Established associations of obesity with chronic diseases, such as cardiovascular disease, hypertension, diabetes, and 13 types of cancer [2,3,4,5,6], underscore the urgency to confront obesity’s widespread impact on metabolic health.

Our previous work in the Multiethnic Cohort Study showed significant differences in abdominal fat amount among racial/ethnic groups that correlated with disparities in the prevalence of metabolic syndrome, type-2 diabetes, and other obesity-related diseases. Notably, compared to African Americans, whites, Latinos, Native Hawaiians, and Japanese Americans exhibited higher amounts of visceral and liver fat, in that order, for a given level of total adiposity [7]. 

The role of visceral adipose tissue (VAT) in cancer disparities is especially intriguing, given its heightened metabolic activity, the differential propensity of racial/ethnic groups to cumulate VAT [7], and evidence of its association with breast cancer [8]. Visceral fat nests around internal abdominal organs, while liver fat localizes within the liver’s cellular structure. When fat builds up in the liver, it can directly influence portal circulation by releasing free fatty acids, leading to the elevation of triglycerides and setting the stage for non-alcoholic fatty liver disease (NAFLD) and the more comprehensive syndrome, Metabolic Dysfunction-associated Fatty Liver Disease (MAFLD) [9,10,11]. It has been suggested that VAT’s proposed link to cancer might relate to its promotion of insulin resistance, inflammation, and the release of associated bioactive compounds [12,13]. These bioactive molecules, such as the adipokines leptin and adiponectin, coupled with inflammatory cytokines like TNF-α and IL-6, have critical functions in cell communication and metabolic activities [14,15]. When secreted from VAT, these compounds may intensify chronic inflammation and metabolic imbalances, which might play a part in the initiation and promotion of several health conditions, including tumor development.

Continuous daily energy restriction (DER) and reduction of adiposity have potential long-term health benefits, including reducing the risk of chronic diseases [16]. Intermittent energy restriction (IER) has shown advantages over a standard DER, including the observation that it may be an easier diet to follow and maintain [17,18]. IER has been shown to be effective at reducing weight in normal weight and overweight/obese men, women, adolescents, and children. Moreover, IER has been found to improve insulin sensitivity, vital for diabetes prevention [19], and positively affect cardiovascular health by reducing blood pressure and improving lipid profiles [20]. It also demonstrates potential cognitive benefits and might influence lifespan [21]. However, adequate evidence of the efficacy of IER, especially among diverse populations, is lacking, as are specific data on its impact on VAT. Additional metabolic measurements are also desirable to provide detailed evidence of underlying mechanisms.

Intermittent Energy Restriction (IER) includes various fasting strategies, each with specific characteristics. Time Restrictive Eating (TER) confines food intake to a definitive window each day, often 8–10 h, promoting fasting for the remaining 14–16 h. However, to date, TRE has not demonstrated consistently strong results in terms of adherence and metabolic health outcomes compared to other IER methods [22]. The 5:2 diet involves usual eating five days per week while significantly reducing calorie intake on two consecutive days. Alternate Day Fasting (ADF) alternates between days of regular eating and days of minimal calorie intake (about 25% of normal). We selected the 5:2 diet due to its promising ability to boost adherence and metabolic health [23]. Its structured yet adaptable method of energy restriction offers a practical substitute for everyday calorie cuts, harmonizing well with the nutritional advantages of the Mediterranean diet [24].

Endorsement for the Mediterranean (MED) diet as a health-promoting diet is growing, with evidence pointing to its effectiveness in diminishing VAT and liver fat accumulation [25,26]. Drawing inspiration from the culinary traditions of coastal Mediterranean regions, such as those in parts of Greece, Spain, and Southern Italy, this dietary approach stresses the importance of plant-based foods [27,28,29]. This includes a wide variety of fruits, vegetables, grains, and legumes. Olive oil is typically the staple for cooking and dressings, with a preference for fish and poultry over red meats. Yogurt and cheese are the dairy mainstays, consumed in moderation. Many have heralded the health advantages of MED, particularly for heart health and certain malignancies. In parallel to the MED diet, (IER) is gaining interest as a viable method to counteract visceral and aberrant fat, particularly when related to liver conditions [30,31,32]. Even though preliminary data suggest benefits from blending the MED diet and IER to combat ectopic fat, initial studies often had relatively small or homogenous participant groups [33]. 

Our group conducted a randomized active-comparator pilot study (the Healthy Diet and Lifestyle Study or HDLS) from 2016–2017 to show the feasibility and effect that an IER + MED diet, compared to the often-used DASH diet, could have on VAT when followed for 12 weeks [33]. Participants in the IER + MED group successfully completed 90.6% of their IER days. No significant adverse effects were reported. On a scale of 1 (low) to 10 (high), diet compliance scores for IER + MED averaged 7.7, while DASH scores were slightly lower at 6.63. The IER + MED group experienced a significantly greater decrease in daily energy intake and greater adherence to prescribed macronutrient percentages than the DASH group. Most notably, the IER + MED group demonstrated a more substantial decrease in several anthropometric and DXA fat measures as compared to the DASH group, including VAT. This was observed even after accounting for the concurrent change in overall adiposity, where the reduction in VAT, adjusted for total fat mass change, was larger in the IER + MED group. Additionally, the IER + MED group exhibited almost double the reduction in other anthropometric measures compared to the DASH group. Roughly 73% of IER + MED participants lost at least 5% of their weight compared to 32% for DASH. Post-intervention, the IER + MED group maintained their body weight at the 6-month visit, while the DASH group saw an increase. The majority of participants in both groups continued with their prescribed diets, with the IER + MED group showing a longer adherence post-study. 

Our primary focus in the Healthy Diet and Lifestyle Study 2 (HDLS2) is to compare the efficacy of IER vs. DER in reducing visceral adiposity and improving metabolic biomarkers, particularly those related to obesity-related diseases. The HDLS2 study compares the IER + MED with DER + MED diets in a 24-week randomized trial among an expected 312 participants aged 35–69. The study incorporates the same behavioral component in both study arms, involving personalized nutrition counseling as well as a moderate-level exercise program. The protocol is described herein. 

## 2. Specific Aims/Outcomes

### 2.1. Aim 1: Effect of Intervention on Visceral and Ectopic Fat

To investigate the comparative effects of two dietary interventions, IER + MED and DER + MED, encompassing equivalent overall energy restrictions and analogous dietary compositions, on visceral and liver fat reduction.

Primary Outcomes:

- Intra-Abdominal VAT Volume: Assessed via abdominal MRI to detect changes attributable to the dietary interventions. Three-dimensional (3D) volumetric assessment of VAT over the abdominal region is used to improve the robustness of the VAT endpoint [34]. The approach is based on 3D volumetric Dixon fat images and automated software that performs localization and segmentation to produce VAT, subcutaneous adipose tissue (SAT), and Abdominal Adipose Tissue (AAT) volume outputs over the abdominal region of interest [35]. VAT refers to the fat deep within the abdominal cavity that surrounds organs, whereas SAT accumulates under the skin. AAT encompasses both VAT and SAT within the abdominal region.
- Liver Fat Percentage: This is quantified using MRI proton density fat fraction (PDFF) and magnetic resonance spectroscopy (MRS) to measure alterations in liver fat content. MRI-PDFF and MRS are MR-based noninvasive quantitative imaging modalities enabling liver fat assessment with high accuracy, repeatability, and reproducibility. They have emerged as a surrogate for liver biopsy in clinical trials [36].
- Relative VAT: Adjustments in VAT measures made in relation to total fat mass as determined by whole-body DXA.
- Total Fat Mass (FM): Comprehensive assessment via whole-body DXA to gauge overall changes in total adiposity.

### 2.2. Aim 2: Effect of Behavioral Factors on Adherence to Intervention

Examine how behavioral factors influence participant adherence to dietary interventions, which can provide insights for designing more effective dietary guidelines and interventions. 

In addition to these two aims, we also include an in-person visit at Week 48 to evaluate the post-intervention maintenance of the intervention and its effects on DXA estimation of VAT [37], overall DXA-total fat mass (FM), and body weight, which will be compared among participants and to the corresponding measures taken at the end of the intervention at Week 24. The precision and accuracy of DXA for estimating VAT are considered reliable [37,38].

## 3. Materials and Methods

### 3.1. Study Design and Participants

HDLS2 is being conducted at the University of Hawai’i Cancer Center (UHCC) and the University of Hawai’i (UH) Magnetic Resonance Imaging (MRI) Research Center. This is a randomized trial comparing two diets over 24 weeks: the IER + MED vs. the DER + MED. The trial inclusion and exclusion criteria are presented in Table 1. 

Recruitment from the general population began in April 2022 through advertising that includes Facebook/email/flyers, media exposure, community presentations, workplace presentations, and referrals from participants and clinical partners. Specifically, we are seeking Oʻahu residents of East Asian, European White, Native Hawaiian, Pacific Islander, or Filipino ancestry, aged 35 to 69, who are slightly or more than slightly overweight and have not smoked in the last 2 years. Participants from other racial or ethnic groups are not eligible for the study. Participants should be willing to improve their eating and exercise habits and committed to following a 24-week diet and physical activity regimen. Ultimately, 312 study participants will be recruited and randomized. Joining the study comes at no cost, with benefits including support from a dietitian, access to the latest science to improve health, and compensation for time and travel. 

Participants are screened for eligibility through a preliminary phone interview, covering aspects like age, ethnicity, BMI, smoking and alcohol intake status, and compatibility for MRI scans. Approximately 436 potential participants are expected to be scheduled for an eligibility visit at the UHCC Clinic, with around 30% projected to be ineligible due to factors like insufficient visceral fat level or abnormal blood biochemistry. The informed consent process explains potential risks associated with the study, such as discomfort from venipuncture or exposure to a small amount of radiation from DXA scans. Premenopausal women must agree to a pregnancy test prior to DXA and MRI scans, which needs to be negative. Once consent is obtained, DXA and MRI compatibility is also checked through questionnaires during recruitment (and before each MRI scan) to ensure participant safety. A clinic visit is then scheduled to further assess eligibility. A more in-depth medical history and a blood chemistry panel are performed to assess general health, an anthropometric examination, and a DXA and abdominal MRI scan to further assess the study body composition requirements (Table 1) and provide baseline measures.

This trial is monitored by the Data and Safety Monitoring Committee of the University of Hawaii Cancer Center (UHCC). The behavioral intervention used in this trial has been evaluated and determined to only have minimal risk for participants by our Institutional Review Board. 

### 3.2. Randomization and Masking

At the end of the first study visit to the clinic (Week 0), participants are randomized to either the IER or DER group. Randomization is stratified by gender, age (35–54 years and 55–69 years for men and pre- and post-menopausal for women), and VAT level (≥100 cm^2^ and <100 cm^2^). The randomization procedure uses sequentially numbered opaque envelopes, each containing an assignment to one of the two dietary interventions. Recruitment and clinic staff remain blinded to the randomization assignment. The intervention staff (dietitians) are blinded to assessment measures of the study participants, apart from their baseline demographic information.

### 3.3. Timing of Outcome Measurements

As shown in Table 2, exposure and outcome measurements assess the impact of dietary interventions on participants throughout the study. Primary outcomes that include changes in VAT volume, liver fat percentage, and total fat mass are assessed using MRI and DXA scans at baseline, Week 12, and Week 24 to capture mid-intervention and post-intervention effects. Secondary outcomes, such as dietary adherence and metabolic biomarkers, are evaluated through self-reported questionnaires and blood tests at the same time points. Metabolic parameters such as ALT, AST, glucose, insulin, leptin, total cholesterol, HDL, LDL, and triglycerides will be measured to assess changes in metabolic biomarkers.

### 3.4. Diet and Physical Activity Interventions

Dietary plans are being individualized for each participant based on their sex, baseline weight, height, and age. Participants in the DER + MED group follow the MED diet continuously, incorporating a 20% energy restriction each day. This restriction is measured against their individual baseline energy requirements or a standard daily caloric intake level that will sustain the baseline weight. The Mediterranean (MED) diet intervention provides low carbohydrate intake (45% energy), with 25% energy from protein and 30% energy from fat.

The IER + MED group is prescribed a 70% energy restriction over two consecutive days each week while following the same macronutrient distribution outlined in the MED diet plan. On the remaining five days, they follow the euenergetic MED diet at the daily caloric intake level that will sustain the baseline weight. This pattern of 70% restriction on 2 of 7 days per week results in an overall energy restriction of 20%. Both dietary plans emphasize foods rich in Monounsaturated Fatty Acids (MUFAs). Intended fat intake targets include 15% energy from MUFA, 8% from Polyunsaturated Fatty Acids (PUFA), and 7% from saturated fat. The protocol allows up to 4% of carbohydrate energy to be allocated to alcohol intake to accommodate light, non-habitual drinkers. Moderate alcohol consumption, defined as up to 10 g per day, does not markedly influence hepatic fat accumulation, suggesting that such levels of intake can be compatible with the successful application of IER and DER strategies. [39].

The implementation of the intervention aligns with the recommendation of conducting at least 14 in-person or web-based sessions led by trained experts for effective obesity management [40]. The counseling sessions focus on both diet and physical activity. No food is provided in HDLS2. Participants choose their foods in both diet groups. Clear instructions are provided during face-to-face consultations with research dietitians on following the assigned diet. Detailed information on portion sizes, recipes, and meal plans, is provided for at-home adherence. Participants are also provided with a local restaurant eating guide, Mediterranean recipes for all meals, and Hawaiian Island-Asian-inspired Mediterranean recipes to better align with local diets. A research dietitian meets with each participant through phone calls or video meetings weekly for the first four weeks following randomization and then every other week until Week 24. A detailed schema illustrating the study design and timeline is provided as Supplementary Materials (Figure S1).

**Figure 1:**
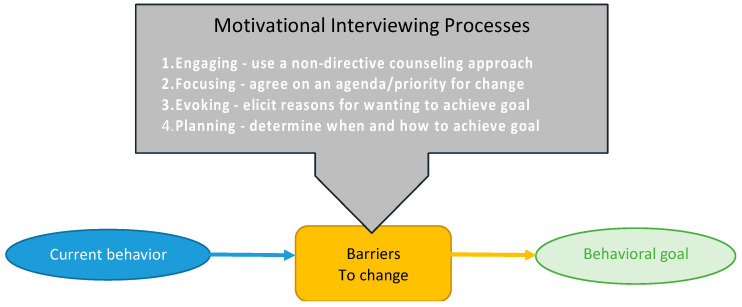
Motivational Interviewing Process.

Dietitians are using behavioral techniques to support diet adherence in both groups. Assigned dietitians contact participants one week after randomization to confirm diet initiation, assess understanding, and offer troubleshooting advice. Research dietitians were trained on the motivational interviewing process, originally developed to treat drug addiction, adapted from the Body and Soul Trial (Figure 1) [41,42,43]. Using theoretical constructs from Social Cognitive and Self-Determination theory to enhance self-efficacy, the research dietitian acts as a problem-solving partner by listening reflectively and offering self-motivational statements in a participant-centered, goal-oriented process to overcome obstinacy and create lasting solutions [44,45]. Building participant skills through motivational interviewing can help them address other situations influencing long-term commitment to diet-related behavior change [17]. Enhancing problem-solving skills through motivational interviewing can also help build social support and aid in addressing toxic food environments [17,46,47].

All participants are asked to increase physical activity, with a program of walking for at least one hour, five days a week. The IER + MED group is encouraged to walk only on non-IER days. Both groups receive resistance bands, providing medium resistance, at the first visit when randomization occurs. A descriptive handout is provided with the resistance bands that contain specific exercises, such as bicep curls and seated leg press exercises. Resistance training involving 3 sets of 8–10 repetitions is prescribed for two nonconsecutive days per week.

### 3.5. Adherence to the Intervention

Adherence to the assigned dietary intervention is being monitored through dietary records for 4 consecutive days using a mobile app called Technology Assisted Dietary Assessment (TADA) that works with most smartphones to capture before and after photos of what participants eat and drink [48]. In addition to photos before and after meals, the TADA app also records time, location, and context [49]. The mobile food record (mFR™) assesses energy, fats (MUFA, PUFA, and saturated fats), carbohydrates, protein, dietary fiber, and alcohol. For the IER + MED group, participants are asked to record their two consecutive IER days on Days 2 and 3 of the four-day mFR™. The DER + MED participants are asked to include one weekend day in their four-day mFR™. The mFR™ data are collected over a 4-day period at baseline, then at Weeks 5, 9, 11, 17, 21, 23, and 47 (Table 2). Maintenance of lifestyle changes is monitored through follow-up surveys and mFRs™. Seven 4-day mFRs™ are collected after baseline, allowing for a thorough assessment of the sustainability of diet-induced alterations. We also assess changes in physical activity by using a questionnaire assessing the average minutes of walking or alternate activity during each counseling session. To ensure participants are supported in maintaining their dietary changes, a research dietitian provides ongoing counseling, initially weekly and then biweekly, through various forms of communication, including phone and video meetings. A final session occurs in Week 48.

To objectively monitor participants’ adherence to exercise recommendations, we evaluate physical activity in Weeks 0, 11, and 23. All participants will follow a standardized physical activity regimen, including walking and resistance exercises. Physical activity will be assessed using questionnaires and ActiGraph™ monitors (ActiGraph™ GT3X+, Pensacola, FL, USA) worn for consecutive days during these weeks, with data processed using ActiLife™ Software version 6.13.6. Physical activity data will be compared between the two study groups and considered as potential confounders in the analysis. This assessment involves wearing an activity monitor (ActiGraph™) on the wrist for one week, complemented by a sleep questionnaire reflecting the time spent in bed and noting any time up during the night. During their first clinic visit, participants are taught how to use the accelerometer. Participants are instructed to wear the monitor continuously, with exceptions being not to wear it in salt water and to avoid wearing it in fresh water for durations exceeding 30 min or at depths greater than 3 feet. Before the designated wear period, they are mailed the accelerometer, instructions, and a return mailer. Standard manufacturer protocols provide activity measurements via ActiLife™ Software version 6.13.6. Our physical activity measurements are based on ActiGraph’s™ validated and transparent activity counting algorithms [50]. Data must align with self-reported wear and sleep times to be valid, requiring at least four days/nights of sufficient wear time. Activity intensities are categorized based on count rates, and the daily average of each level (e.g., sedentary, moderate) is used in the analysis.

### 3.6. Data Analysis

A data management system was created for the HDLS2 study based on strategies from the pilot HDLS. The system manages participant recruitment, milestone tracking, specimen handling and storage, and data entry. A detailed review and cleaning of data is performed soon after collection. Aggregation of all collected data, including DXA and MRI measures, biomarkers, and dietary intake data, will be performed prior to data analysis.

The study will assess the effects of dietary interventions on the main endpoints, such as visceral and liver fat, and explore the factors driving dietary adherence. Under Specific Aim 1, an intent-to-treat and a difference-in-differences approach to compare changes in adiposity, at Weeks 12 and 24 between treatment arms, using linear mixed models and Wald tests [51]. We will compare the effectiveness of the intervention by subgroup (sex, age, and VAT level, defined as in the randomization) to gain insight into its generalizability. Similar analyses will be conducted with the metabolic parameters endpoints, including ALT, AST, glucose, insulin, leptin, total cholesterol, HDL, LDL, and triglycerides.

In Specific Aim 2, we will identify, for each treatment arm, the predictors of dietary adherence among participant characteristics and psychosocial and behavioral factors using machine-learning approaches, such as LASSO regression, that allow for consideration of many independent variables. This analysis will provide insights into the factors influencing adherence to each dietary intervention.

Our sample size of 312 randomized participants (assuming a ~16% drop-out rate to yield 260 participants completing the 6-month intervention) with an equal split across the two study arms will provide 80% power to detect small to medium effect sizes (d = 0.35) [52] in primary and secondary outcomes, including visceral fat and body weight, with an alpha (two-sided) of 0.05 (d = 0.49) for analysis of men and women separately. Power is modestly attenuated after multiple comparison control, with (d = 0.54) with alpha = 0.005 (Bonferroni-corrected for 100 tests).

## 4. Results

Participant enrollment commenced in April 2022 and will end in September 2025. No results are yet available.

## 5. Discussion

Obesity, specifically concerning visceral adipose tissue (VAT), is a public health challenge, often addressed through DER strategies. The long-term efficacy of these methods is limited due to issues like weight regain. IER is emerging as an alternative strategy and is showing effectiveness in weight loss [53]. Studies such as Harvie et al. (2013) [54], Ash et al. (2003) [55], and Varady et al. (2011) [20] showed that IER can lead to significant weight loss, averaging 0.2–0.8 kg/week, comparable to DER [56]. In addition, studies like Arguin et al. (2012) [57] and Williams et al. (1998) [58] have reported outcomes on body composition, showing reductions in fat mass and waist circumference.

In comparative studies against more traditional dieting approaches, various forms of IER, including alternate-day fasting and time-restricted feeding, have been analyzed by Hutchison et al. (2019) [59], Liu et al. (2019) [60], and Bowen et al. (2018) [61]. These studies demonstrated their effectiveness in generating weight loss and metabolic improvements over periods ranging from 8 to 52 weeks [53]. The present research contributes to the understanding of IER’s role in obesity management, particularly in relation to VAT reduction. However, considering its implications for public health, the long-term efficacy and specific impacts on VAT will warrant more extensive research into IER’s potential in obesity and VAT management [53].

Our methodology stands out due to its comprehensive and innovative approach due to: (1) the precise quantification of fat using MRI and abdominal DXA, providing precise assessments of visceral and liver fat changes; (2) the incorporation of behavioral support techniques, such as motivational interviewing with a dietitian, to enhance practical application and adherence to dietary protocols; and (3) the continuous and detailed monitoring of participants through mobile technology, capturing dietary and lifestyle changes over time for a robust longitudinal analysis. Our inclusive recruitment strategy, designed to cover a diverse range of racial/ethnic backgrounds, aims to ensure that the study’s findings are widely applicable and representative of different population groups.

However, the study is not without limitations. Self-reported data on diet and exercise, even with the aid of technology, could introduce an element of reporting bias. However, there is no expectation that this bias would differ between study arms. The extensive nature of the intervention may also lead to a greater-than-expected participant dropout, impacting the study’s statistical robustness. Additionally, the relatively strict exclusion criteria, although necessary, might limit the generalizability of the findings. These considerations will be important in interpreting the potential impact and scope of the study’s findings.

We believe the HDLS2 study will provide valuable insights into effective diet patterns for reducing visceral and liver fat, as well as for improving metabolic biomarkers. It incorporates a comprehensive approach, considering not just the dietary and physical activity components but also the behavioral aspects of lifestyle change, which are crucial for the success of any dietary intervention. However, attention needs to be given to the integrity of the data analysis and interpretation of results, ensuring the limitations are adequately considered.

## Figures and Tables

**Table 1:** Inclusion and Exclusion Criteria for Study Participation.

Inclusion Criteria	Exclusion or Deferral Criteria
Age 35 to 69 years	Pregnancy Contraindications to MRI (e.g., pacemaker, metal implants, claustrophobia)
BMI between 25 and 40 kg/m^2^	Surgical history involving the rectum, colon, small intestine, or limb amputation
Residence within a 20-mile radius of the UHCC	Inability to engage in at least one hour of daily exercise (walking)
Of Native Hawaiian, Other Pacific Islander, Japanese, Chinese, Korean, Filipino, or European/white ancestry	Individuals receiving insulin for Type 1 or Type 2 diabetes
Non-smoker	Receiving medication or hormones for thyroid treatment
No serious underlying health issues	
Non-drinker or low habitual alcohol intake (<15 drinks/week for men, <10 drinks/week for women)	Men taking anti-androgen medications
Fully vaccinated against COVID-19	Substantial recent weight change (≥20 pounds/10 kg in <6 months)
Normal blood chemistry profile	Received chemotherapy or radiation therapy in the past six months
VAT area at L4/L5 ≥90 cm^2^ for men, ≥80 cm^2^ for women (estimated by DXA)	
	Using steroid hormones (Cortisone, Prednisone, Methylprednisone) or weight-loss prescriptions (Lorcaserin, Orlistat, Phentermine, Qsymia) in the past six months

**Table 2:** Timeline of intervention activities for HDLS2.

Week	Activity	Outcome Assessments	Mobile Diet Records (mFR™)	Dietary Counseling Sessions
Week 0	Baseline Visit	Blood draw, DXA, MRI, Questionnaires, Physical Exam, Clinical Measurements	4 days	1 in-person session
Weeks 1–11	Remote Counseling		4 days (Weeks 5, 9, and 11)	7 by phone or video
Week 12	Mid-Study Visit 2 (Clinic Visit)	Blood draw, DXA, MRI, Questionnaires, Physical Exam, Clinical Measurements		1 in-person session
Weeks 13–23	Remote Counseling		4 days (Weeks 17, 21, and 23)	5 by phone or video
Week 24	End of Intervention Visit (Clinic Visit)	Blood draw, DXA, MRI Questionnaires, Physical Exam, Clinical Measurements		1 in-person session
Weeks 25–47	Maintenance (No Specific Activity)	Clinical Follow-up, Questionnaires	4 days (Week 47)	
Week 48	Follow-up Visit (Clinic Visit)	Blood draw, DXA, Questionnaires, Physical Exam, Clinical Measurements		1 in-person follow-up session

## Data Availability

Data will be made available upon reasonable request.

## Supplementary Materials

The following supporting information can be downloaded at: https://www.mdpi.com/article/10.3390/nu16101478/s1, Figure S1: Schema of the Healthy Diet & Lifestyle 2 Protocol (HDLS2).
