# Supplementary figures and images for: Effect of Intermittent vs. Continuous Energy Restriction on Visceral Fat: Protocol for The Healthy Diet and Lifestyle Study 2 (HDLS2)
Source: Nutrients. 2024 May 14;16(10):1478. doi: 10.3390/nu16101478 (PMC11123735; doi:10.3390/nu16101478)

## Schema – The Healthy Diet & Lifestyle 2 Protocol (HDLS2)

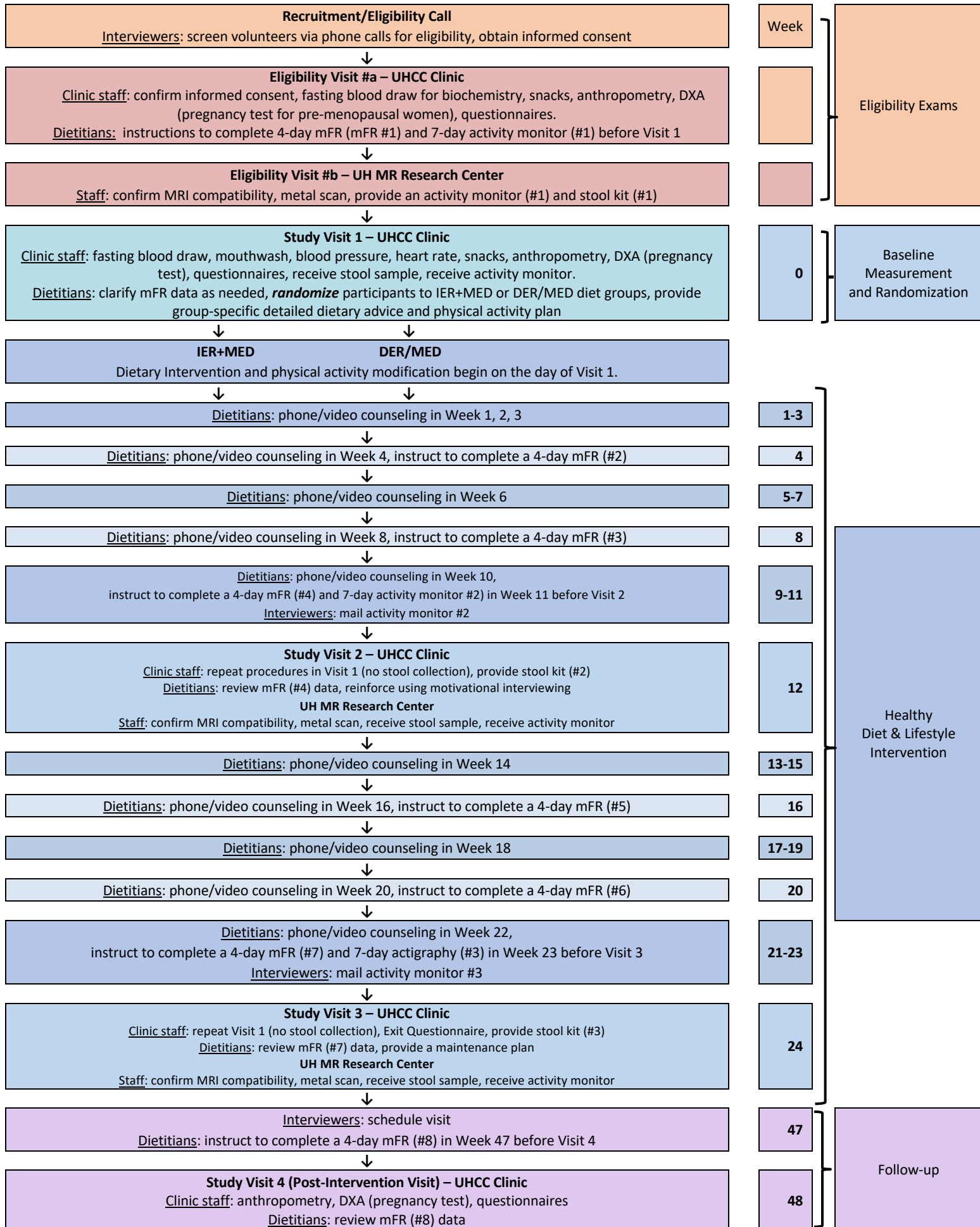

Supplement: Supplementary file 1 [file nutrients-16-01478-s001.zip › nutrients-2952341-supplementary.pdf]
